# Who receives an MCI diagnosis?

**DOI:** 10.1093/geroni/igaf122.1911

**Published:** 2025-12-31

**Authors:** Elyse Couch, Munachimso Ugoh, Lauren Thomas, Emmanuelle Belanger

**Affiliations:** Brown University, Providence, Rhode Island, United States; Brown University, Providence, Rhode Island, United States; Brown University, Providence, Rhode Island, United States; Brown University, Providence, Rhode Island, United States

## Abstract

Mild cognitive impairment (MCI) is a key risk factor for future dementia and represents an opportunity for early engagement in treatment and decision-making. Although MCI affects more people than dementia, little is known about MCI diagnosis rates. This study aimed to determine the proportion of people with MCI who received a formal diagnosis and identify patient characteristics associated with an MCI diagnosis in a nationally representative sample of Medicare beneficiaries in the United States. To achieve this aim, we used data from the National Health and Aging Trends Study (NHATS) linked with Medicare claims data. We included beneficiaries enrolled in the 2015 round of NHATS, who developed symptoms of cognitive impairment consistent with MCI according to a validated algorithm of NHATS measures of cognition, and were continuously enrolled in Medicare fee-for-service. Beneficiaries were followed up until 2022. Beneficiaries with diagnosed MCI were identified in Medicare claims data using ICD-9 and ICD-10 codes for MCI. Univariate and multivariate logistic regressions were used to identify patient characteristics associated with diagnosed MCI. Of 2.7 million continuously enrolled Medicare beneficiaries with symptoms of cognitive impairment consistent with MCI, only 10.6% had a diagnosis of MCI recorded in claims. Multivariate analyses found the odds of diagnosed MCI were higher among women, beneficiaries with a bachelor’s degree or higher, and lower among beneficiaries who attended doctor visits alone. The findings of this study suggest that MCI is significantly under-diagnosed. Future research and policy initiatives are needed to increase MCI diagnosis rates.

